# A systematic review with meta-analysis on the efficacy of 0.01% atropine eyedrops in preventing myopia progression in worldwide children’s populations

**DOI:** 10.3389/fphar.2025.1497667

**Published:** 2025-05-22

**Authors:** Pierluigi Navarra, Luca Buzzonetti, Valentina Amico, Melina Cro, Bruno Federico

**Affiliations:** ^1^ Section of Pharmacology, Department of Healthcare Surveillance and Bioethics, Catholic University Medical School, Fondazione Policlinico Universitario A. Gemelli-Istituto di Ricovero e Cura a Carattere Scientifico, Rome, Italy; ^2^ Ophthalmology Unit, Bambino Gesù Children’s Hospital, IRCCS, Rome, Italy; ^3^ Medivis S.r.l., Catania, Italy; ^4^ Department of Human Sciences, Society and Health, University of Cassino and Southern Lazio, Cassino, Italy

**Keywords:** atropine, myopia, children, meta-analysis, systematic review

## Abstract

**Introduction:**

Atropine eyedrops have long been used off-label to prevent myopia progression in children, and many clinical trials have been published on this topic in the past 30 years. Trials initially tested doses ranging from 0.01% to 1%, but more recently, the interest has turned to low doses, mainly 0.01%. Moreover, the first studies were carried out in Asian populations, but the number of trials conducted in other geographical areas has rapidly increased from 2020 onward. This meta-analysis was aimed at summarizing the evidence on the efficacy of 0.01% atropine eyedrops on the reduction of myopia progression, also comparing study findings from different parts of the world.

**Methods:**

Data were obtained from PubMed, Scopus and Web of Science databases from 1 January 1 2020 to 31 July 2024. Randomized controlled trials involving children receiving 0.01% atropine eyedrops for at least 1 year were included. Heterogeneity was quantified by Q, H, and I2 statistics, and a meta-analysis was performed using a random effect model. The risk for bias was assessed using the Cochrane Collaboration (Chapter 6) aspects of bias scale.

**Results and discussion:**

The primary outcomes were the differences in spherical equivalent refractive errors and axial length at baseline and after 12 months of treatment with 0.01% atropine eyedrops or placebo. Eleven studies involving 2,046 children (1,172 receiving 0.01% atropine eyedrops and 874 receiving placebo) were included. Atropine was significantly more effective than placebo, with an average reduction of 0.16/year (95% CI: 0.11–0.22) and −0.07/year (95% CI: −0.09 to −0.05) in spherical equivalent refractive errors and axial length, respectively. The efficacy of 0.01% atropine eyedrops vs. placebo was maintained in a subpopulation of subjects after 24 months of treatment. We found no difference in atropine efficacy between Southeast Asian populations (1,063 children, 52%) and populations in various other countries (983 children, 48%).

## Introduction

Atropine has long been used off-label to prevent myopia progression in children, despite the fact that, until today, the exact mechanism of action remains unclear (Upadhyay and Beuerman, 2020). The first reports of atropine use in myopia date to the 1980s–1990s (Yen et al., 1989; Shih et al., 1999). Initially, the range of atropine doses investigated in randomized controlled trials (RCTs) and cohort studies was quite large, spanning from 1% to 0.01% eyedrop concentration, with the latter being relatively less used; it noteworthy that in a large meta-analysis published in 2017, only a single study testing 0.01% atropine eyedrops (not an RCT) was included in the review, which involved 3,137 subjects (Gong et al., 2017). Indeed, initial dose–response studies (Chia et al., 2012; Yam et al., 2019) indicated that the efficacy of atropine in preventing myopia progression is dose-dependent, with the highest efficacy observed at 1% concentration (Chia et al., 2012). However, it was also increasingly clear that the rate and severity of adverse events are directly related to atropine dose (Sun et al., 2023), which makes the use of high-dose atropine (0.5%–1%) unsuitable in the setting of long-term treatments. Moreover, a rebound effect has been described in association with treatment stop, whose extent appeared to be concentration-dependent (Yam et al., 2022). Therefore, in the last few years, the use of lower atropine doses (mostly 0.01%–0.02%) became prevalent in clinical trials (Yam et al., 2019; Wei et al., 2020; Hieda et al., 2021; Saxena et al., 2021; Lee et al., 2022; Chia et al., 2023; Hansen et al., 2023; Repka et al., 2023; Zadnik et al., 2023; Loughman et al., 2024; Wang et al., 2024).

A matter of concern with the use of atropine lies in the inconsistencies across diverse ethnicities, which require further validation (Zhang et al., 2024). In fact, most spontaneous studies carried out in the period 2000–2020 were conducted in Chinese and other Asian populations (Gong et al., 2017) because the so-called “myopia epidemics” (Dolgin, 2024) first showed in these geographic areas. However, in the period from 2020 onward, the number of RCTs carried out outside Asian regions has increased (Lee et al., 2022; Hansen et al., 2023; Repka et al., 2023; Zadnik et al., 2023; Loughman et al., 2024), so that it is now possible to compare the effects of atropine treatments among trials carried out in different world areas.

In the present study, we carried out a meta-analysis of results from RCTs investigating the efficacy of long-term treatments with 0.01% atropine eyedrops in preventing myopia progression in children. We aimed to summarize the existing evidence about the efficacy of 0.01% atropine eyedrops in this clinical setting and its efficacy throughout populations of different geographic areas.

## Materials and methods

### Patient intervention comparison outcome (PICO) framework

The research question was formulated using the PICO framework as follows: P (children with myopia) – I (daily administration of 0.01% atropine eyedrops) – C (daily administration of placebo eyedrops) – O (changes at 12 months and 24 months in spherical equivalent refractive error (SER) and axial length (AL))

### Data source and search strategy

A comprehensive literature search was performed from 1 January 1 2000 to 31 July 31 2024. We used the PubMed database (which provides access, in addition to Medline, to some other databases, including Index Medicus and PMC citations), Scopus, and Web of Science. The keywords for searching included “atropine,” “myopia,” and “randomized controlled trial.” Supplementary Appendix S1 contains the search string for each bibliographic database.

**FIGURE 1:**
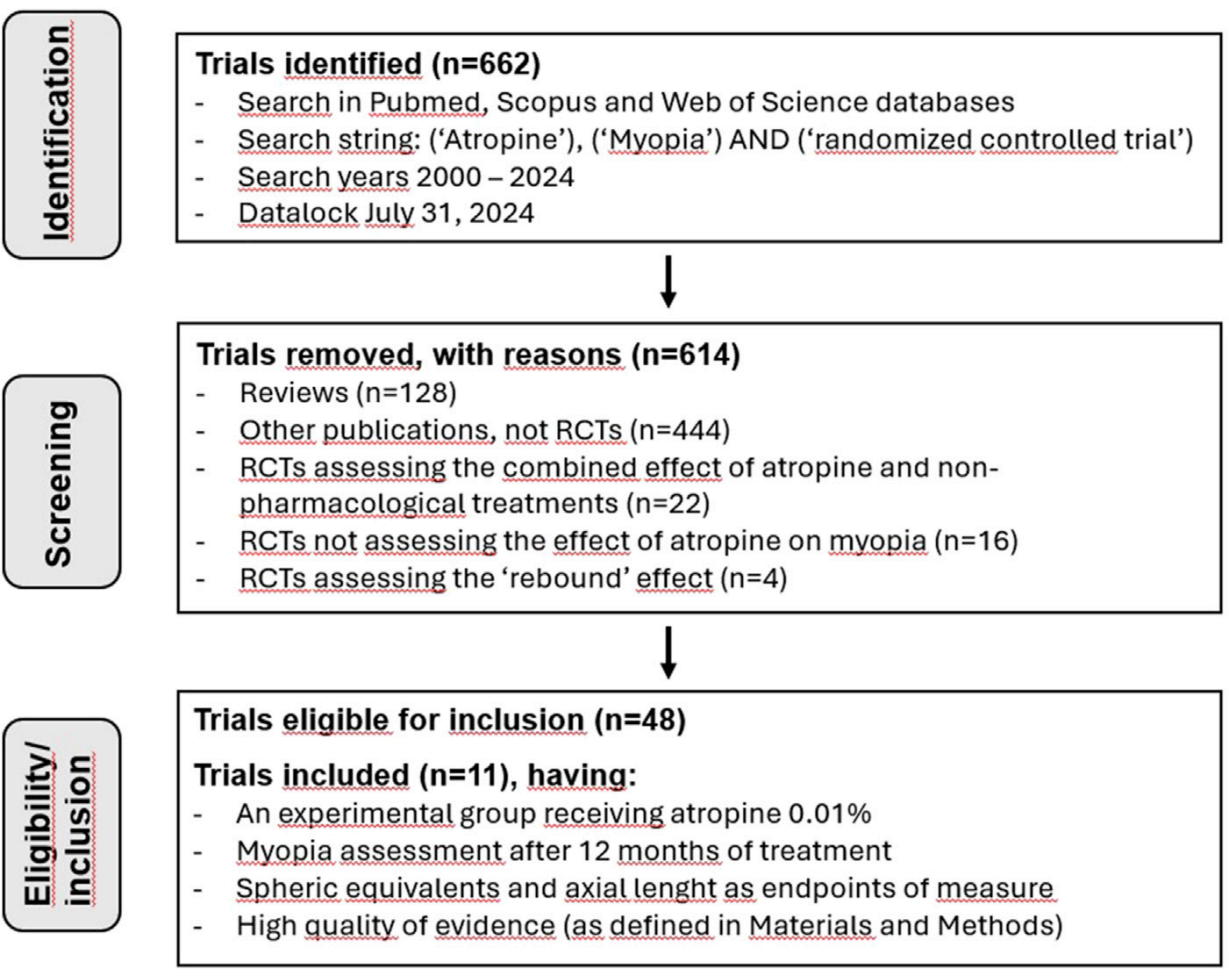
Flow chart of trial search and selection.

Studies were included in the analysis if they included the following criteria: 1) RCTs showing a control group receiving placebo and an experimental group receiving 0.01% atropine eyedrops; 2) myopia assessment after 12 months of treatment; 3) SER and AL assessed as endpoints. SER was calculated by adding the sum of the sphere power with half of the cylinder power assessed under cycloplegic condition, Whereas AL values were obtained by measuring the distance from the front to the back of the eye; 4) high quality of the evidence (see below, Quality of studies). Exclusion criteria included 1) reviews; 2) types of study that were not RCTs (cohort studies, case reports, single-group studies, etc.); 3) animal studies; 4) repeated or overlapping publications; 5) studies reporting the combined effects of non-pharmacological treatments; 6) studies on atropine reporting other endpoints; 7) studies assessing the rebound effect of atropine. The process of study selection is shown in Figure 1. If more than one publication reported the same data, only the first publication in chronological order was used in the analysis. This is the case, for instance, of Liang et al. (2023), which was excluded from the analysis because it contained the same data reported by Yam et al. (2019).

### Data extraction

The information was reviewed and extracted by three authors independently using predefined data summary lists. Two of the three authors resolved any disagreements regarding the data extraction. The detailed information collected was summarized in a table containing the name of the first author, year of publication, study country, sample size in the atropine and placebo groups, age of children, SER and AL mean values at baseline, and months of follow-up (Table 1). According to the country where the selected studies have been conducted, the study populations could be divided into two subgroups, namely, “Southeast Asia” (studies carried out in India, Singapore, China, and Japan) and “Rest of the world” (studies carried out in Europe, Australia, and the United States).

**TABLE 1:** Characteristics of the studies included in the review.

Author	Year	Country	A^a^	P^a^	Age (years)^b^	SER (D)^b^	AL (mm)^b^	Follow-up (months)
Chia et al.	2023	Singapore	25	26	8.9	−3.7	24.8	12
Hansen et al.	2023	Europe	32	32	9.4	−3.0	24.6	12
Hieda et al.	2021	Japan	85	86	9.0	−2.9	24.4	12
Lee et al.	2022	Australia	104	49	11.7	−3.3	24.6	24
Loughman et al.	2023	Europe	167	83	11.8	−3.3	24.9	24
Repka et al.	2023	United States	125	62	10.1	−2.8	24.4	24
Saxena et al.	2021	India	50	50	10.7	−3.6	N.A.	12
Wang et al.	2024	China	200	100	9.1	−2.3	24.5	12
Wei et al.	2020	China	110	110	9.6	−2.6	24.6	12
Yam et al.	2019	China	110	111	8.3	−3.8	24.8	12
Zadnik et al.	2023	Europe/United States	164	165	8.9	−2.7	24.4	36

^a^ Number of randomized patients.

^b^ Mean values at baseline.

### Quality of studies

The quality of RCTs was assessed using the risk of bias tool from the Cochrane Collaboration (Higgins et al., 2011). Evaluation criteria included 1) random sequence generation (selection bias); 2) allocation concealment (selection bias); 3) masking of participants and researchers (performance bias); 4) masking of outcome assessment (detection bias); 5) incomplete outcome data (attrition bias), 6) selective reporting (reporting bias), and other bias. Each risk of bias was categorized as either low, unclear, or high risk after assessment (Table 2). Trials were considered to have low-quality evidence (and then excluded from the final analysis) if they had three or more bias items ranked “high risk” and/or “some concern,” according to Higgins et al. (2011).

**TABLE 2:** Risk of bias assessment.

Author	Year	D1	D2	D3	D4	D5	D6	D7	Overall
Chia et al.	2023								
Hansen et al.	2023								
Hieda et al.	2021								
Lee et al.	2022								
Loughman et al.	2023								
Repka et al.	2023								
Saxena et al.	2021								
Wang et al.	2024								
Wei et al.	2020								
Yam et al.	2018								
Zadnik et al.	2023								

D1 Random sequence generation (selection bias)

D2 Allocation concealment (selection bias)

D3 Blinding of participants and personnel (performance bias)

D4 Blinding of outcome assessment (detection bias)

D5 Incomplete outcome data (attrition bias)

D6 Selective reporting (reporting bias)

D7 Other sources of bias (other bias)

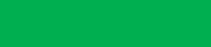
 Low risk

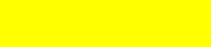
 Some concerns

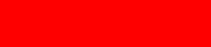
 High risk

### Statistical analysis

As reported above, the outcome variables of the analyses were SER and AL. For each outcome, we computed the mean difference (MD) in 12-month changes between 0.01% atropine eyedrops (A) and placebo (P). We used MD and its standard error if they were available from the publication. If these were not reported but mean (m) and standard deviation (s) of 12-month change were instead available, we computed the mean difference as $MD=m_{A}-m_{P}$ , and its standard error was defined as $SE\left(MD\right)=\sqrt{\frac{s_{A}^{2}}{n_{A}}+\frac{s_{P}^{2}}{n_{P}}}$ .

The random effects meta-analysis was performed using the DerSimonian–Laird method to estimate tau2. Higgins I2 was computed to evaluate the degree of heterogeneity (Egger et al., 2022). A subgroup meta-analysis was carried out to assess if patient characteristics (patients from Southeast Asia vs. patients from other countries) modified the treatment effect. A separate analysis was performed of the studies that reported 24-month changes in SER and AL.

We performed Egger’s regression test to investigate the presence of publication bias. In addition, funnel plots that contrasted effect size versus the standard error of the estimate were visually inspected.

## Results

Table 1 shows the characteristics of the trials included in this meta-analysis. Eleven trials met the inclusion and exclusion criteria, including a total of 2,046 children: of these, 1,172 subjects received 0.01% atropine eyedrops once daily before sleep, and 874 subjects received the corresponding placebo. All studies were published between 2018 and 2023. Five of 11 trials were carried out in non-Asian regions, including Europe, the United States, and Australia; 983 subjects were included in these trials (592 receiving 0.01% atropine eyedrops and 321 receiving placebo), which represented 48% of the whole population included in the present meta-analysis. Moreover, four trials (Lee et al., 2022; Repka et al., 2023; Zadnik et al., 2023; Loughman et al., 2024) had SER and AL assessed after 24 months of treatment and could be analyzed for the effects of atropine after 24 months of treatment as well.

Table 2 presents the risk of bias assessment conducted according to (Higgins et al., 2011). One trial was excluded (Jethani, 2022), as it raised “some concerns” about selection, performance, and detection bias and “high risk” about attrition bias. The selected trials are generally high-quality studies.

**FIGURE 2:**
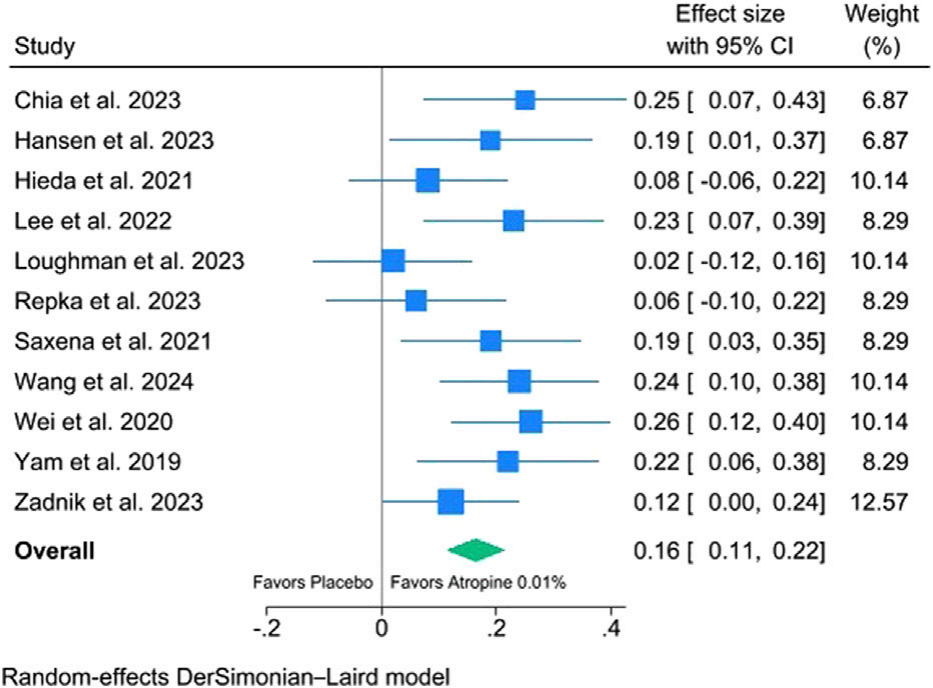
Meta-analysis of 12-month changes in spherical equivalent refractive error (SER) of 0.01% atropine eyedrops versus placebo.

The results of the meta-analysis on 12-month changes in SER of 0.01% atropine eyedrops versus placebo are shown in Figure 2. The figure shows that 0.01% atropine eyedrops are significantly more effective than placebo in reducing myopia progression, with an average reduction in SER of 0.16/year (95% CI: 0.11–0.22). There is a very small degree of heterogeneity (I2 = 24.4%).

**FIGURE 3:**
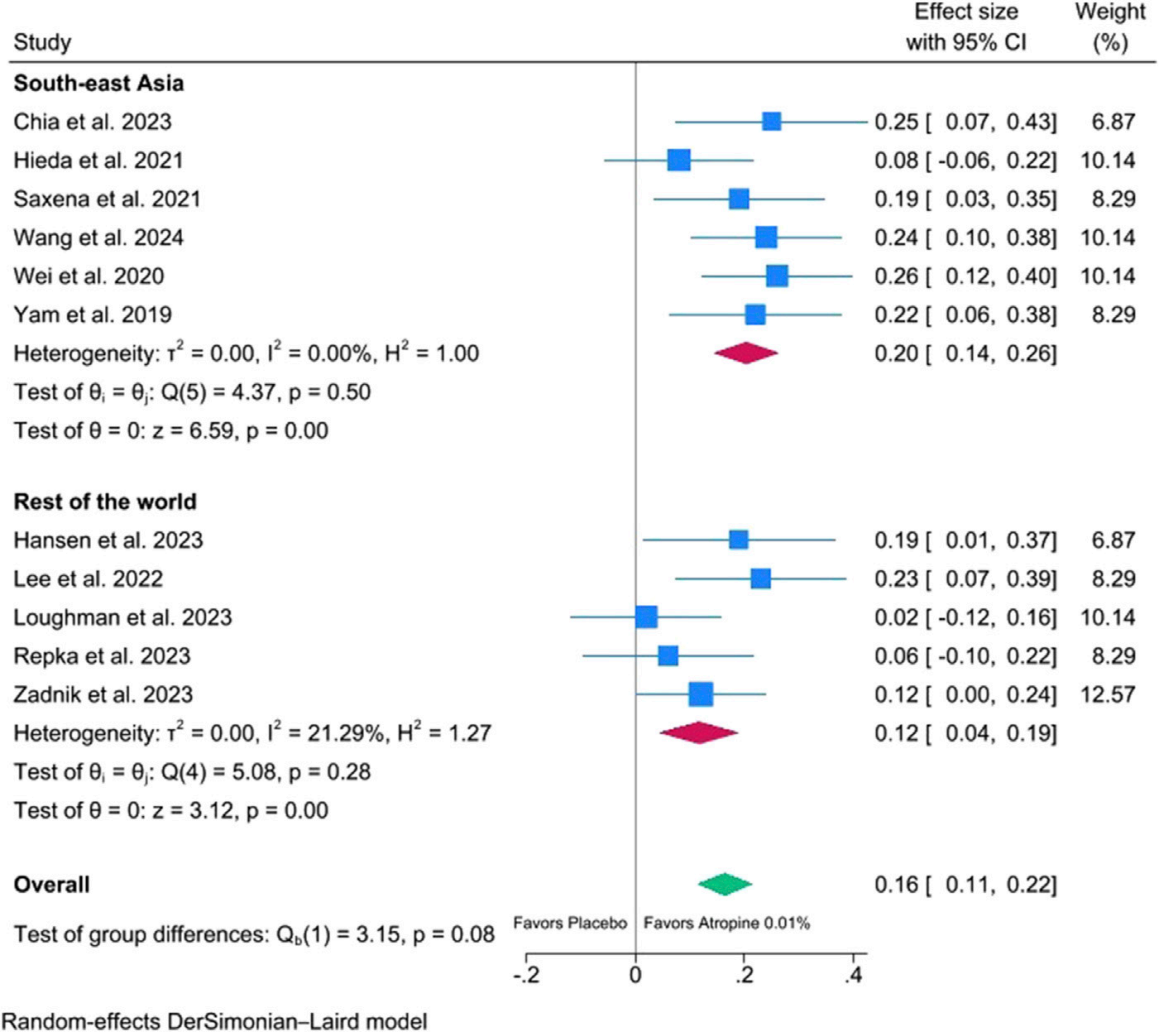
Meta-analysis of 12-month changes in spherical equivalent refractive error (SER) of 0.01% atropine eyedrops versus placebo stratified by continent.

Figure 3 shows the meta-analysis of 12-month changes in SER of 0.01% atropine eyedrops versus placebo stratified by geographical region (Southeast Asia versus rest of the world). The effect of 0.01% atropine eyedrops on SER is significant in both Asian and non-Asian populations. There is a trend, albeit not significant, to a difference between Asia (effect size: 0.20; 95% IC: 0.14–0.26) and other geographical areas (effect size: 0.12; 95% IC: 0.04–0.19).

**FIGURE 4:**
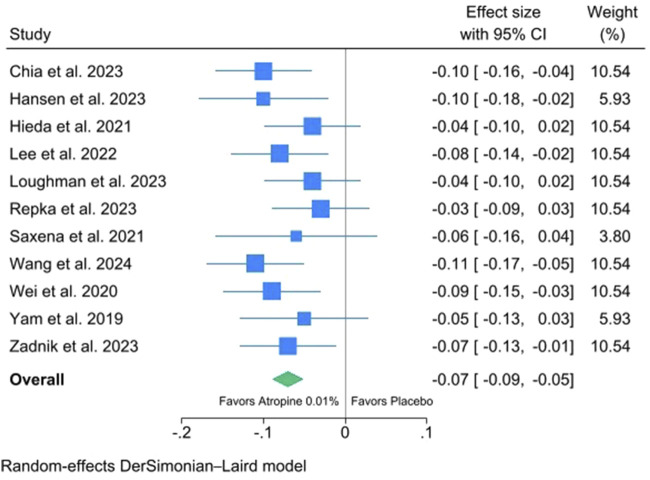
Meta-analysis of 12-month changes in axial length (AL) of 0.01% atropine eyedrops versus placebo.

The results of a meta-analysis on 12-month changes in AL of 0.01% atropine eyedrops versus placebo are shown in Figure 4. Similar to SER, 0.01% atropine eyedrops are significantly more effective than placebo in reducing myopia progression, with an average reduction in AL of −0.07/year (95% CI: −0.09 to −0.05). Moreover, there is no evidence of heterogeneity, and all studies provide a remarkably similar effect of 0.01% atropine eyedrops on AL.

**FIGURE 5:**
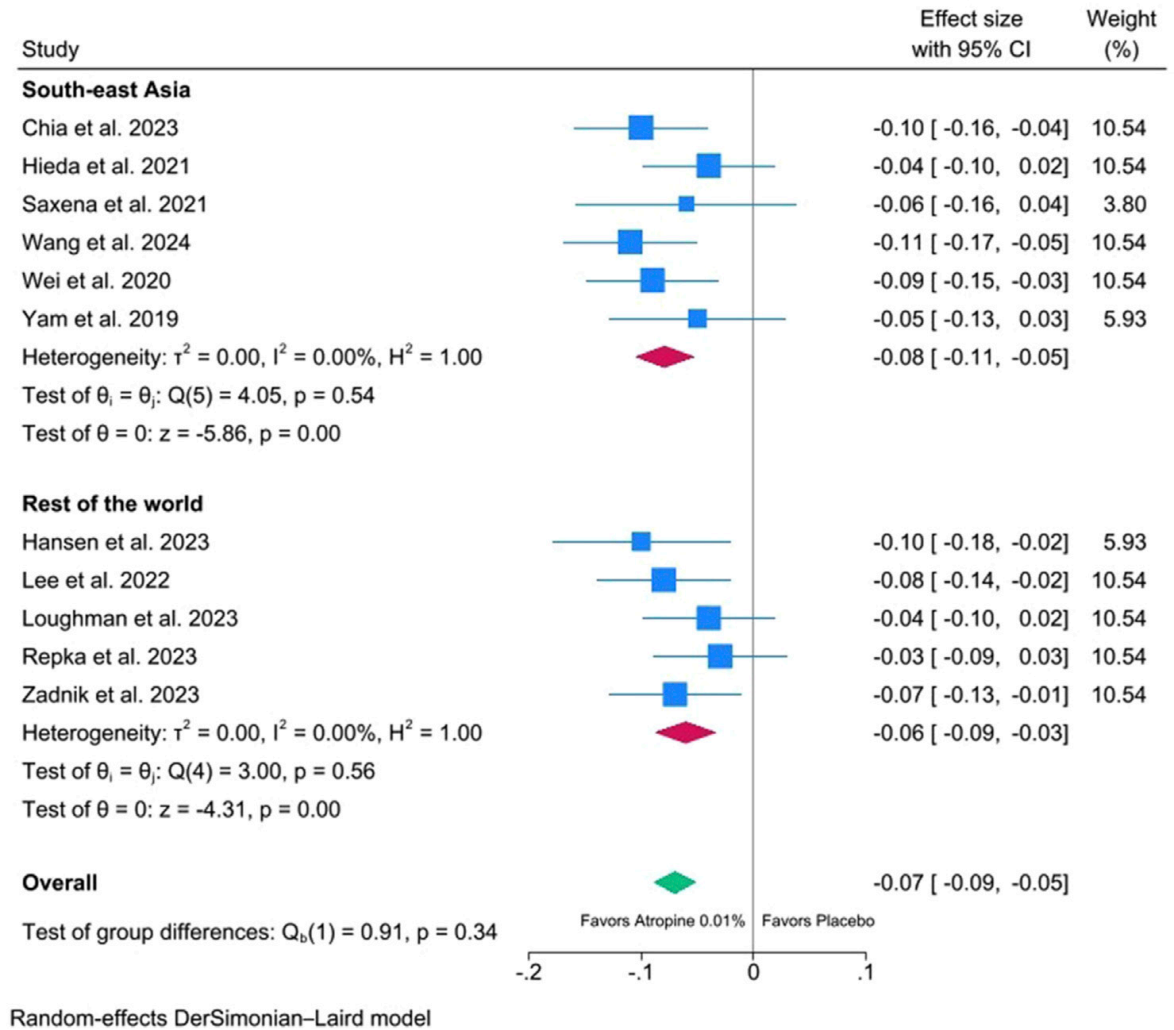
Meta-analysis of 12-month changes in axial length (AL) of 0.01% atropine eyedrops versus placebo stratified by continent.

Figure 5 shows the meta-analysis of 12-month changes in AL of 0.01% atropine eyedrops versus placebo stratified by geographical region (Southeast Asia versus other countries). Similar to SER, the effect of 0.01% atropine eyedrops on AL is significant in both Asian and non-Asian populations.

**FIGURE 6:**
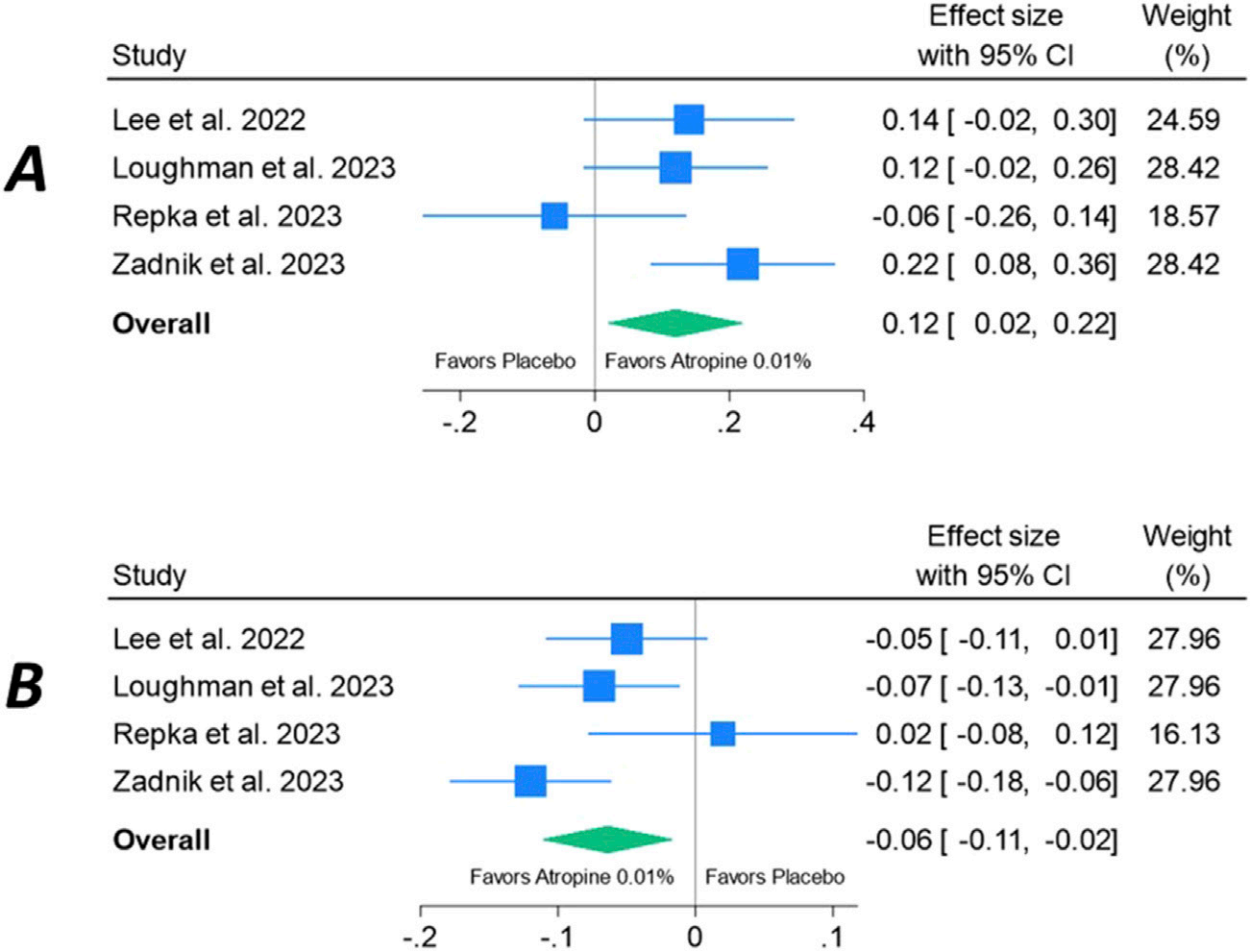
Meta-analysis of 24-month changes in spherical equivalent refractive error **(A)** and axial length **(B)** of 0.01% atropine eyedrops versus placebo.

Figure 6 shows the meta-analysis of 24-month changes in SER (Panel A) and AL (Panel B) of 0.01% atropine eyedrops versus placebo. A favorable effect of atropine vs. placebo is maintained, with overall effect sizes of 0.12 on SER (95% CI: 0.02–0.22) and 0.06 on AL (95% CI: −0.11 to −0.02), which are similar the results at 12 months. For both endpoints, the results of the study by Repka et al. (2023) clearly differ from the other studies.

## Discussion

Myopia is a common refractive error influenced by various factors such as genetics, environmental exposures, and lifestyle habits (Lawrenson et al., 2023; Eppenberger et al., 2024; Zhang et al., 2024), and it is affecting an increasing number of individuals worldwide (Lawrenson et al., 2023). It is estimated that in 2050, 50% of the world population will be myopic (Nucci et al., 2023). A high risk of ocular diseases such as retinal detachment, glaucoma, and myopic maculopathy, which is one of the actual leading causes of low vision and blindness in developed countries, is correlated with high myopia (Flitcroft, 2012; Tideman et al., 2016; Lawrenson et al., 2023).

Because myopia is usually detected in children before 10 years of age, and its prevalence could record a fast progression after the age of six, an increasing need exists for therapeutic strategies to slow myopia progression in childhood (Lawrenson et al., 2023). Some authors reported that the mean annual myopia progression rate in children was approximately half a diopter in Europe (−0.55D) and slightly higher in Asia (−0.82D) (Donovan et al., 2012).

Three broad therapeutic options are currently considered for slowing myopia progression: optical, pharmacological, and environmental (Lawrenson et al., 2023), and the most commonly used topical pharmacological intervention for myopia progression control is atropine. Recently, some authors observed a synergistic effect in slowing myopia progression, combining an optical component, which involves peripheral defocus spectacles or contact lenses, and the biological component, represented by atropine. Together, they seem to act more efficaciously than when used separately. Nucci et al. (2023) reported the most successful results combining 0.01% atropine eyedrops with defocus incorporated multiple segments spectacles compared with separate efficacy. On the same line of evidence, Erdinest et al. (2024) observed that the combination of 0.05% atropine eyedrops and peripheral defocus soft contact lenses effectively controls myopia progression in children.

The exact mechanism of action for atropine in reducing myopia progression is still unknown (Upadhyay and Beuerman, 2020). One pathway is the inhibition of accommodative function via muscarinic receptors. There are several hypotheses regarding atropine’s mode of action with sites of action in the sclera, retinal pigment epithelium, and choroid, but up to now, no consensus has been reached (Jawaid et al., 2024).

Although initial evidence suggested that atropine shows a dose-dependent effect in reducing myopia progression in children (Song et al., 2011), two more recent meta-analyses demonstrated that no difference could be observed between various doses of atropine in the range of 0.01%–1% (Huang et al., 2016; Gong et al., 2017). Conversely, a recent meta-analysis has clearly shown that the incidence of adverse events related to atropine increases in a dose-dependent manner (Sun et al., 2023). These authors also showed that there was no difference in the rate of adverse events for low-dose atropine between Asian and White children (Sun et al., 2023). Overall, this evidence led to preferring using 0.01% in spontaneous RCTs in the last few years (Lawrenson et al., 2023). We have detected this trend in our analysis because eight of the nine papers selected have been published in the last 4 years.

Long-term side effects of atropine use in children have been reported in the function of concentration percentage: changes in accommodation amplitude, changes in pupil size, and photophobia. Yam et al. (2019) reported that few patients require hospitalization. There was one case each of gastroenteritis, influenza, and asthmatic attack in the 0.05% atropine eyedrop group. In the 0.025% atropine eyedrops group, one participant had gastroenteritis, one participant had pneumonia, one participant had elective circumcision surgery, and two participants had influenza. In the 0.01% atropine eyedrops group, one participant had a lip injury requiring surgical repair, one participant had influenza, and one participant had a distal radius fracture requiring plaster casting. In the placebo group, two participants had influenza (Yam et al., 2019).

Real-world evidence also showed the efficacy of low-dose atropine in slowing myopia progression. Usmani et al. (2023) reported that 0.01% atropine eyedrops were effective and well tolerated within a real-time clinical setting during the COVID-19 pandemic despite regular follow-ups being difficult to maintain. Sacchi et al. (2019) published a retrospective study that reflects real-life clinical practice; these authors also observed that 0.01% atropine eyedrops were effective in European myopic patients.

In conclusion, the present meta-analysis work produced two main findings: *i*) 0.01% atropine eyedrops are significantly better than placebo in reducing myopia progression (assessed through SER and AL measurements) in 12-month treatments; *ii*) the same is true in both Asian and non-Asian populations. Some considerations can be drawn about the first conclusion; first, the average effect size is rather limited, which might raise some concern about the clinical relevance and usefulness of long-term atropine treatments (Moriche-Carretero et al., 2021). In any case, the limited size effect of the treatment strengthens the need for a very high tolerability, especially considering the long duration of treatment. Another consideration concerns the differences between the two endpoints investigated in the study; it would appear that AL shows slightly higher consistency across the studies, suggesting its larger use in patient monitoring in the setting of clinical routine.

As far as the comparison between different geographic areas, namely, Asia vs. Rest of the world, the observation that 0.01% atropine eyedrops are effective regardless of geographical and ethnic differences in the study populations is a relevant new finding, which may encourage more widespread use of atropine to prevent myopia progression in children.

## Data Availability

The original contributions presented in the study are included in the article/Supplementary Material; further inquiries can be directed to the corresponding author.
