# Supplementary material for: A systematic review with meta-analysis on the efficacy of 0.01% atropine eyedrops in preventing myopia progression in worldwide children’s populations
Source: Front Pharmacol. 2025 May 22;16:1497667. doi: 10.3389/fphar.2025.1497667 (PMC12137072; doi:10.3389/fphar.2025.1497667)
Supplement: Supplementary file 1 [file DataSheet2.docx]

Re-analysis April 2025

. * meta-analysis for SER

. meta set ser_mean ser_es, random(dlaird) studylabel(author)

Meta-analysis setting information

Study information

No. of studies: 11

Study label: author

Study size: N/A

Effect size

Type: <generic>

Label: Effect size

Variable: ser_mean

Precision

Std. err.: ser_es

CI: [_meta_cil, _meta_ciu]

CI level: 95%

Model and method

Model: Random effects

Method: DerSimonian–Laird

. meta forestplot, noohetstat noohomtest noosigtest nullrefline(favorsleft("Favors Placebo", size(small)) favorsright("Favors Atr

> opine 0.01%", size(small))) saving(ser.gph, replace)

Effect-size label: Effect size

Effect size: ser_mean

Std. err.: ser_es

Study label: author

file ser.gph saved

. meta summarize

Effect-size label: Effect size

Effect size: ser_mean

Std. err.: ser_es

Study label: author

Meta-analysis summary Number of studies = 11

Random-effects model Heterogeneity:

Method: DerSimonian–Laird tau2 = 0.0018

I2 (%) = 24.40

H2 = 1.32

-----------------------------------------------------------------------

Study | Effect size [95% conf. interval] % weight

---------------------+-------------------------------------------------

Chia et al. 2023 | 0.250 0.074 0.426 6.87

Hansen et al. 2023 | 0.190 0.014 0.366 6.87

Hieda et al. 2021 | 0.080 -0.057 0.217 10.14

Lee et al. 2022 | 0.230 0.073 0.387 8.29

Loughman et al. 2023 | 0.020 -0.117 0.157 10.14

Repka et al. 2023 | 0.060 -0.097 0.217 8.29

Saxena et al. 2021 | 0.190 0.033 0.347 8.29

Wang et al. 2024 | 0.240 0.103 0.377 10.14

Wei et al. 2020 | 0.260 0.123 0.397 10.14

Yam et al. 2019 | 0.220 0.063 0.377 8.29

Zadnik et al. 2023 | 0.120 0.002 0.238 12.57

---------------------+-------------------------------------------------

theta | 0.164 0.113 0.215

-----------------------------------------------------------------------

Test of theta = 0: z = 6.29 Prob > |z| = 0.0000

Test of homogeneity: Q = chi2(10) = 13.23 Prob > Q = 0.2112

. meta summarize, leaveoneout

Effect-size label: Effect size

Effect size: ser_mean

Std. err.: ser_es

Study label: author

Leave-one-out meta-analysis summary Number of studies = 11

Random-effects model

Method: DerSimonian–Laird

-------------------------------------------------------------------

Omitted study | Effect size [95% conf. interval] p-value

---------------------+---------------------------------------------

Chia et al. 2023 | 0.158 0.104 0.211 0.000

Hansen et al. 2023 | 0.163 0.107 0.218 0.000

Hieda et al. 2021 | 0.174 0.120 0.227 0.000

Lee et al. 2022 | 0.158 0.104 0.213 0.000

Loughman et al. 2023 | 0.179 0.132 0.226 0.000

Repka et al. 2023 | 0.173 0.121 0.226 0.000

Saxena et al. 2021 | 0.162 0.106 0.218 0.000

Wang et al. 2024 | 0.156 0.102 0.210 0.000

Wei et al. 2020 | 0.153 0.101 0.205 0.000

Yam et al. 2019 | 0.159 0.104 0.214 0.000

Zadnik et al. 2023 | 0.171 0.114 0.227 0.000

---------------------+---------------------------------------------

theta | 0.164 0.113 0.215 0.000

-------------------------------------------------------------------

. meta funnelplot, mlabel(author)

Effect-size label: Effect size

Effect size: ser_mean

Std. err.: ser_es

Model: Common effect

Method: Inverse-variance

. meta bias, egger detail traditional

Effect-size label: Effect size

Effect size: ser_mean

Std. err.: ser_es

Fixed-effects meta-regression Number of obs = 11

Error: Multiplicative Dispersion phi = 1.30

Method: Inverse-variance Model F(1,9) = 1.16

Prob > F = 0.3102

------------------------------------------------------------------------------

_meta_es | Coefficient Std. err. t P>|t| [95% conf. interval]

-------------+----------------------------------------------------------------

_meta_se | 3.136796 2.916986 1.08 0.310 -3.461886 9.735477

_cons | -.0704945 .2181911 -0.32 0.754 -.564077 .423088

------------------------------------------------------------------------------

Test of residual homogeneity: Q_res = chi2(9) = 11.72 Prob > Q_res = 0.2295

Regression-based Egger test for small-study effects

Fixed-effects model

Method: Inverse-variance

H0: beta1 = 0; no small-study effects

beta1 = 3.14

SE of beta1 = 2.917

t = 1.08

Prob > |t| = 0.3102

.

. * subgroup analysis

. meta summarize, subgroup(continent)

Effect-size label: Effect size

Effect size: ser_mean

Std. err.: ser_es

Study label: author

Subgroup meta-analysis summary Number of studies = 11

Random-effects model

Method: DerSimonian–Laird

Group: continent

-----------------------------------------------------------------------

Study | Effect size [95% conf. interval] % weight

---------------------+-------------------------------------------------

Group: South-east A~a|

Chia et al. 2023 | 0.250 0.074 0.426 6.87

Hieda et al. 2021 | 0.080 -0.057 0.217 10.14

Saxena et al. 2021 | 0.190 0.033 0.347 8.29

Wang et al. 2024 | 0.240 0.103 0.377 10.14

Wei et al. 2020 | 0.260 0.123 0.397 10.14

Yam et al. 2019 | 0.220 0.063 0.377 8.29

|

theta | 0.203 0.143 0.264

---------------------+-------------------------------------------------

Group: Rest of the ~d|

Hansen et al. 2023 | 0.190 0.014 0.366 6.87

Lee et al. 2022 | 0.230 0.073 0.387 8.29

Loughman et al. 2023 | 0.020 -0.117 0.157 10.14

Repka et al. 2023 | 0.060 -0.097 0.217 8.29

Zadnik et al. 2023 | 0.120 0.002 0.238 12.57

|

theta | 0.117 0.044 0.191

---------------------+-------------------------------------------------

Overall |

theta | 0.164 0.113 0.215

-----------------------------------------------------------------------

Heterogeneity summary

-----------------------------------------------------------------------------

Group | df Q P > Q tau2 % I2 H2

---------------+-------------------------------------------------------------

South-east A~a | 5 4.37 0.497 0.000 0.00 1.00

Rest of the ~d | 4 5.08 0.279 0.002 21.29 1.27

---------------+-------------------------------------------------------------

Overall | 10 13.23 0.211 0.002 24.40 1.32

-----------------------------------------------------------------------------

Test of group differences: Q_b = chi2(1) = 3.15 Prob > Q_b = 0.076

.

. meta forestplot, noohetstat noohomtest noosigtest nullrefline(favorsleft("Favors Placebo", size(small)) favorsright("Favors Atr

> opine 0.01%", size(small))) saving(ser_continent.gph, replace) subgroup(continent)

Effect-size label: Effect size

Effect size: ser_mean

Std. err.: ser_es

Study label: author

file ser_continent.gph saved

.

. * meta-analysis for AL

. meta set al_mean al_es, random(dlaird) studylabel(author)

Meta-analysis setting information

Study information

No. of studies: 11

Study label: author

Study size: N/A

Effect size

Type: <generic>

Label: Effect size

Variable: al_mean

Precision

Std. err.: al_es

CI: [_meta_cil, _meta_ciu]

CI level: 95%

Model and method

Model: Random effects

Method: DerSimonian–Laird

. meta forestplot, noohetstat noohomtest noosigtest nullrefline(favorsright("Favors Placebo", size(small)) favorsleft("Favors Atr

> opine 0.01%", size(small))) saving(al.gph, replace)

Effect-size label: Effect size

Effect size: al_mean

Std. err.: al_es

Study label: author

file al.gph saved

. meta summarize

Effect-size label: Effect size

Effect size: al_mean

Std. err.: al_es

Study label: author

Meta-analysis summary Number of studies = 11

Random-effects model Heterogeneity:

Method: DerSimonian–Laird tau2 = 0.0000

I2 (%) = 0.00

H2 = 1.00

-----------------------------------------------------------------------

Study | Effect size [95% conf. interval] % weight

---------------------+-------------------------------------------------

Chia et al. 2023 | -0.100 -0.159 -0.041 10.54

Hansen et al. 2023 | -0.100 -0.178 -0.022 5.93

Hieda et al. 2021 | -0.040 -0.099 0.019 10.54

Lee et al. 2022 | -0.080 -0.139 -0.021 10.54

Loughman et al. 2023 | -0.040 -0.099 0.019 10.54

Repka et al. 2023 | -0.030 -0.089 0.029 10.54

Saxena et al. 2021 | -0.060 -0.158 0.038 3.80

Wang et al. 2024 | -0.110 -0.169 -0.051 10.54

Wei et al. 2020 | -0.090 -0.149 -0.031 10.54

Yam et al. 2019 | -0.050 -0.128 0.028 5.93

Zadnik et al. 2023 | -0.070 -0.129 -0.011 10.54

---------------------+-------------------------------------------------

theta | -0.070 -0.089 -0.051

-----------------------------------------------------------------------

Test of theta = 0: z = -7.21 Prob > |z| = 0.0000

Test of homogeneity: Q = chi2(10) = 7.96 Prob > Q = 0.6324

. meta summarize, leaveoneout

Effect-size label: Effect size

Effect size: al_mean

Std. err.: al_es

Study label: author

Leave-one-out meta-analysis summary Number of studies = 11

Random-effects model

Method: DerSimonian–Laird

-------------------------------------------------------------------

Omitted study | Effect size [95% conf. interval] p-value

---------------------+---------------------------------------------

Chia et al. 2023 | -0.067 -0.087 -0.047 0.000

Hansen et al. 2023 | -0.068 -0.088 -0.049 0.000

Hieda et al. 2021 | -0.074 -0.094 -0.054 0.000

Lee et al. 2022 | -0.069 -0.089 -0.049 0.000

Loughman et al. 2023 | -0.074 -0.094 -0.054 0.000

Repka et al. 2023 | -0.075 -0.095 -0.055 0.000

Saxena et al. 2021 | -0.071 -0.090 -0.051 0.000

Wang et al. 2024 | -0.066 -0.086 -0.045 0.000

Wei et al. 2020 | -0.068 -0.088 -0.048 0.000

Yam et al. 2019 | -0.071 -0.091 -0.052 0.000

Zadnik et al. 2023 | -0.070 -0.090 -0.050 0.000

---------------------+---------------------------------------------

theta | -0.070 -0.089 -0.051 0.000

-------------------------------------------------------------------

. meta funnelplot, mlabel(author)

Effect-size label: Effect size

Effect size: al_mean

Std. err.: al_es

Model: Common effect

Method: Inverse-variance

. meta bias, egger detail traditional

Effect-size label: Effect size

Effect size: al_mean

Std. err.: al_es

Fixed-effects meta-regression Number of obs = 11

Error: Multiplicative Dispersion phi = 0.88

Method: Inverse-variance Model F(1,9) = 0.00

Prob > F = 0.9636

------------------------------------------------------------------------------

_meta_es | Coefficient Std. err. t P>|t| [95% conf. interval]

-------------+----------------------------------------------------------------

_meta_se | .0892473 1.899654 0.05 0.964 -4.20807 4.386564

_cons | -.0730645 .0613725 -1.19 0.264 -.2118987 .0657696

------------------------------------------------------------------------------

Test of residual homogeneity: Q_res = chi2(9) = 7.96 Prob > Q_res = 0.5381

Regression-based Egger test for small-study effects

Fixed-effects model

Method: Inverse-variance

H0: beta1 = 0; no small-study effects

beta1 = 0.09

SE of beta1 = 1.900

t = 0.05

Prob > |t| = 0.9636

.

. * subgroup analysis

. meta summarize, subgroup(continent)

Effect-size label: Effect size

Effect size: al_mean

Std. err.: al_es

Study label: author

Subgroup meta-analysis summary Number of studies = 11

Random-effects model

Method: DerSimonian–Laird

Group: continent

-----------------------------------------------------------------------

Study | Effect size [95% conf. interval] % weight

---------------------+-------------------------------------------------

Group: South-east A~a|

Chia et al. 2023 | -0.100 -0.159 -0.041 10.54

Hieda et al. 2021 | -0.040 -0.099 0.019 10.54

Saxena et al. 2021 | -0.060 -0.158 0.038 3.80

Wang et al. 2024 | -0.110 -0.169 -0.051 10.54

Wei et al. 2020 | -0.090 -0.149 -0.031 10.54

Yam et al. 2019 | -0.050 -0.128 0.028 5.93

|

theta | -0.079 -0.106 -0.053

---------------------+-------------------------------------------------

Group: Rest of the ~d|

Hansen et al. 2023 | -0.100 -0.178 -0.022 5.93

Lee et al. 2022 | -0.080 -0.139 -0.021 10.54

Loughman et al. 2023 | -0.040 -0.099 0.019 10.54

Repka et al. 2023 | -0.030 -0.089 0.029 10.54

Zadnik et al. 2023 | -0.070 -0.129 -0.011 10.54

|

theta | -0.061 -0.088 -0.033

---------------------+-------------------------------------------------

Overall |

theta | -0.070 -0.089 -0.051

-----------------------------------------------------------------------

Heterogeneity summary

-----------------------------------------------------------------------------

Group | df Q P > Q tau2 % I2 H2

---------------+-------------------------------------------------------------

South-east A~a | 5 4.05 0.542 0.000 0.00 1.00

Rest of the ~d | 4 3.00 0.558 0.000 0.00 1.00

---------------+-------------------------------------------------------------

Overall | 10 7.96 0.632 0.000 0.00 1.00

-----------------------------------------------------------------------------

Test of group differences: Q_b = chi2(1) = 0.91 Prob > Q_b = 0.339

. meta forestplot, noohetstat noohomtest noosigtest nullrefline(favorsright("Favors Placebo", size(small)) favorsleft("Favors Atr

> opine 0.01%", size(small))) saving(al_continent.gph, replace) subgroup(continent)

Effect-size label: Effect size

Effect size: al_mean

Std. err.: al_es

Study label: author

file al_continent.gph saved
