# Supplementary material for: A systematic review with meta-analysis on the efficacy of 0.01% atropine eyedrops in preventing myopia progression in worldwide children’s populations
Source: Front Pharmacol. 2025 May 22;16:1497667. doi: 10.3389/fphar.2025.1497667 (PMC12137072; doi:10.3389/fphar.2025.1497667)
Supplement: Supplementary file 2 [file DataSheet1.docx]

**Search strategies**

PubMed

((atropine[Title]) AND (myopi*[Title/Abstract])) AND (trial[Title/Abstract] OR experimental [Title/Abstract] OR controlled[Title/Abstract] OR RCT[Title/Abstract]) NOT(lense*[Title])

Scopus

( TITLE ( atropine ) AND TITLE-ABS-KEY ( randomized AND controlled AND trial ) AND TITLE ( myopia ) ) AND NOT ( lenses )

Web of Science

Atropine myopi* trial (Abstract) not lense*(Title)
